# Sphincter-Preserving Fistulectomy Is an Effective Minimally Invasive Technique for Complex Anal Fistulas

**DOI:** 10.3389/fsurg.2022.832397

**Published:** 2022-03-22

**Authors:** Yinwen Hong, Zhizhong Xu, Ying Gao, Mingming Sun, Yinghui Chen, Ke Wen, Xiaopeng Wang, Xueliang Sun

**Affiliations:** ^1^Department of Obstetrics and Gynecology, Suzhou Hospital of Integrated Traditional Chinese and Western Medicine, Suzhou, China; ^2^Department of Colorectal Surgery, Suzhou TCM Hospital Affiliated to Nanjing University of Chinese Medicine, Suzhou, China

**Keywords:** fistulectomy, ligation of the intersphincteric fistula tract, lift, sphincter-preserving technique, anal fistula

## Abstract

**Background:**

The optimal treatment of complex anal fistulas remains unclear, though many different sphincter-preserving procedures have been described. A minimally invasive technique with a better outcome is desired. The purpose of this study was to present a new technique—sphincter-preserving fistulectomy (SPF) and its clinical outcomes.

**Materials and Methods:**

A retrospective study was performed to compare the efficacy and outcomes of SPF with ligation of the intersphincteric fistula tract (LIFT) in the management of complex anal fistulas in regards to postoperative pain, complications, wound healing time, recurrence, overall success rate, fecal continence function, and quality of life. Continence function was evaluated using the Wexner incontinence scale and anal manometry. The fecal incontinence quality of life (FIQL) scale was used to assess patients' quality of life.

**Results:**

From June 2020 to July 2021, 41 patients with 43 SPF procedures and 35 patients with 35 LIFT procedures were included. Postoperative pain was comparable between two groups. The morbidity rate and the mean wound healing time in the SPF group were lower than those in the LIFT group (2.3% vs. 48.6%, *p* < 0.001; 1.4 ± 0.3 vs. 1.7 ± 0.4 months, *p* = 0.001). At a mean follow-up duration of 11.4 ± 3.5 months in the SPF group and 10.7 ± 4.3 months in the LIFT group, SPF achieved a better overall success rate than LIFT (97.7% vs. 77.1%, *p* = 0.014). Three patients in the SPF group and 4 patients in the LIFT group who all underwent a simultaneous fistulotomy procedure complained new incontinence of flatus. There was no statistical difference between the two groups in regards to the Wexner scores (*p* = 0.790), the maximum resting anal canal pressure (*p* = 0.641), the maximum squeeze pressure (*p* = 0.289), and the FIQL scores including lifestyle (*p* = 0.188), coping (*p* = 0.188), depression (*p* = 0.850), and embarrassment (*p* = 0.910).

**Conclusions:**

SPF is a novel, safe, and effective minimally invasive technique for the management of complex anal fistulas, with a promising success rate and negligible impairment on continence. Future prospective studies are needed to evaluate the long-term outcomes of SPF.

## Introduction

Complex anal fistulas involving higher two-thirds of the external anal sphincter and the levator ani muscle pose a therapeutic challenge to colorectal surgeons due to increased risks of fecal incontinence and recurrence. The strategy of treating complex anal fistulas is to pursue a higher success rate together with maintenance of fecal continence. However, it is hard to have the best of both worlds.

During the past decades, a variety of sphincter-preserving procedures have been presented in attempting to achieve the best balance between a high success rate and perfect fecal continence. Anal fistula plug (AFP) insertion was considered as one of the first-line treatments for complex anal fistulas. Nevertheless, the long-term healing rates of fistulas after AFP were 38–56%, accompanied by impaired function of fecal continence in 26.8% of patients (1, 2). A long fistula tract was demonstrated to be associated with declined success rate and anal function due to potential branch fistulas and abscesses (2). Although fistula injection with fibrin glue, platelet-rich plasma, and mesenchymal stem cells had less adverse effect on fecal continence, the success rates were only 41.7, 48.4, and 50%, respectively (3, 4). Video-assisted anal fistula treatment (VAAFT), an emerging minimally invasive technique, displayed a wide variation in the healing rate of complex anal fistulas, from 22 to 83.3% (5, 6). Multiple fistula tracts and supra/extrasphincteric fistulas were risk factors for recurrence after VAAFT (7). Fistula laser closure (FiLaC®) is another effective sphincter-saving procedure for complex anal fistulas with a long-term success rate of 66.8% (8). A curved tract or a tract with diameters exceeding 4–5 mm might increase the failure rate (8). In general, inadequate closure or elimination of the internal opening was probably the common defect of the above procedures, resulting in unsatisfactory success rates.

Mucosal advancement flap (MAF) was utilized to close the internal opening, after a fistula tract was curetted or cored out. In a meta-analysis study, the pooled success rate of MAF for the treatment of complex anal fistulas was 79%, with a fecal incontinence rate of 13.3% (9). Infection, necrosis, and dehiscence of mucosal flap were major adverse events, resulting in failure or recurrence. Ligation of the intersphincteric fistula tract (LIFT) has been a mainstream procedure for the treatment of complex anal fistulas. The overall success rate of LIFT was 76.5% with fecal continence impairment in 1.4% of patients (10). Horseshoe fistulas and a history of previous fistula surgery were risk factors for failure after LIFT (10). Despite many measures were used to improve the clinical outcomes of LIFT, such as LIFT plus AFP, platelet-rich plasma, or VAAFT, the efficacy was limited (11–13). The costs must be weighed against the benefits.

Fistulectomy had an excellent healing rate of 96.6% in treating simple anal fistulas (14). However, it was not recommended to treat complex anal fistulas because of impairment on anal sphincter. Several modified fistulectomy techniques were described to treat complex anal fistulas in attempting to decrease its impairment on fecal continence. Core-out fistulectomy plus loose-seton showed a short-term healing rate of 87.5% in the treatment of high transsphincteric fistulas, but with keyhole-like deformity in 5% of patients (15). Fistulectomy plus primary sphincteroplasty showed a more excellent success rate of 90.9% in treating high anal fistulas, but with a noteworthy rate of fecal incontinence (16, 17). A modified fistulectomy with an excellent balance between fistula healing and maintenance of fecal continence is anticipated. The aim of the present paper was to describe a novel sphincter-preserving fistulectomy (SPF) technique and evaluate its efficacy in treating complex anal fistulas.

## Materials and Methods

### Study Design

A retrospective study was conducted to review the prospectively collected data on patients who underwent an SPF or LIFT procedure between June 2020 and July 2021. Fistula anatomy was described according to the Parks classification (18). Fistula diagnosis and classification were identified by preoperative MRI. A high transsphincteric fistula was defined as the tract involving more than 50% of the external anal sphincter. Patients with age between 16 and 60 years, high-transsphincteric or suprasphincteric fistulas treated with an SPF or LIFT procedure were included. Exclusion criteria were as follows: (1) patients with age <16 years or >60 years; (2) patients with an intersphincteric, a low transsphincteric, or an extrasphincteric fistula; (3) patients with intestinal diseases, such as inflammatory bowel disease, tuberculosis, and Behcet's disease; (4) patients treated with immunomodulator agents; and (5) patients with uncontrolled chronic diseases or mental diseases. This study was approved by the Ethics Committee of Suzhou TCM Hospital Affiliated to Nanjing University of Chinese Medicine (No. 2017-006). Individual consent for this retrospective analysis was waived.

The data on patient demographics, previous surgical history, length and location of fistulas, MRI and endoscopy findings, operative data, postoperative morbidity, wound healing time, fecal continence function, quality of life scores, and follow-up findings were reviewed. The direct linear distance from the external opening to the anal verge pointing to the internal opening was calculated as the length of a tract. The location of the primary tract was categorized into anterior, lateral, posterior, and semi-horseshoe, based on a linear distribution in the 11–1, 2–4, and 8–10, and 5–7 o'clock in the lithotomy position and a curved distribution. Fecal continence function was evaluated *via* the Wexner incontinence scale and anal manometry, before and 3 months after operation (19). The maximum resting anal canal pressure (MRP) and maximum squeeze pressure (MSP) both in the high-pressure zone were measured with an eight-channel stationary water-perfused manometry catheter (Hefei Aoyuan Technology Development Co. Ltd, Anhui province, China). The fecal incontinence quality of life (FIQL) scale was used to evaluate the quality of life of patients who had a successful fistula closure at the last follow-up and patients who suffered a failure or a recurrence at the last follow-up before reoperation (20).

### Operative Procedure

#### Sphincter-Preserving Fistulectomy

**Figure 1:**
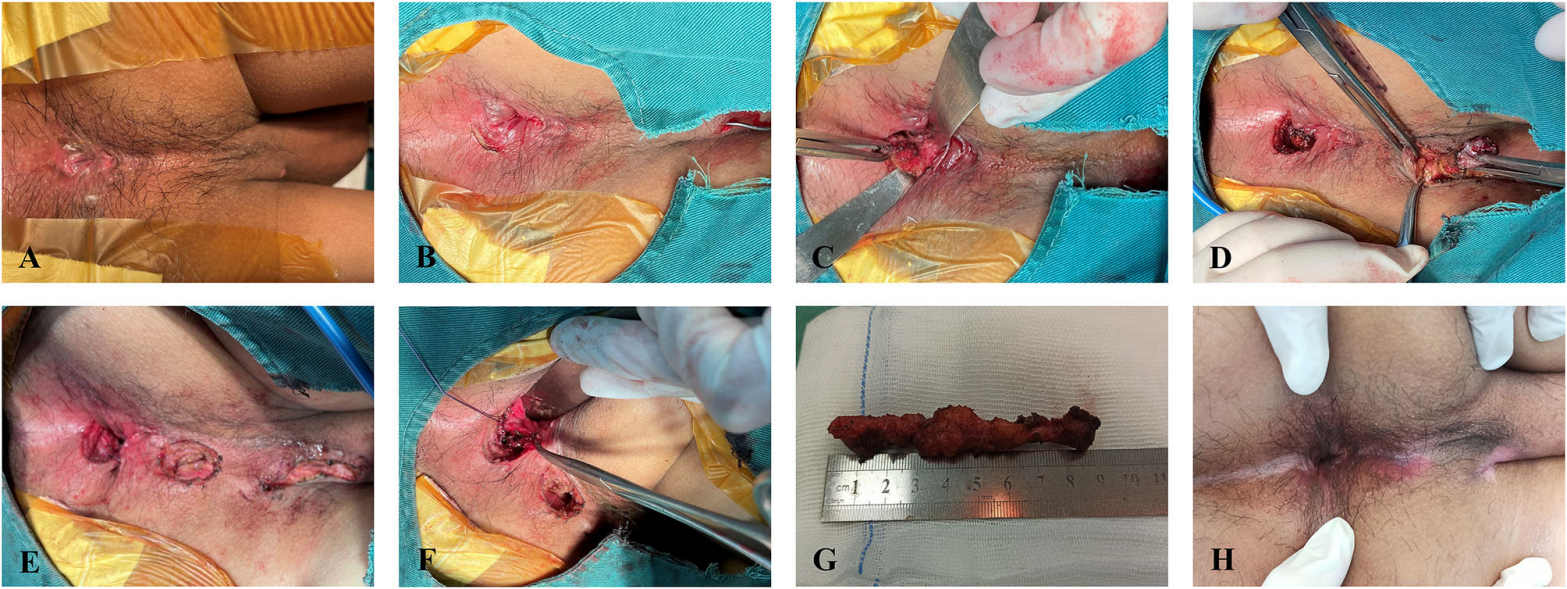
Sphincter-preserving fistulectomy procedure. **(A)** A semi-horse high-transsphincteric fistula. **(B)** An intersphincteric curvilinear incision. **(C)** Dissection of the intersphincteric tract to the external anal sphincter after being cut off adjacent to the internal anal sphincter. **(D)** Coring out the remnant tract. **(E)** Suture repair of the defect on the external anal sphincter after core-out fistulectomy. **(F)** Partial suture of the lateral full-thickness flap after excising the internal opening. **(G)** Excised fistula specimen. **(H)** Normal anal morphology after wound healing.

The evening before surgery, an enema was administered. After spinal anesthesia, the patient was placed in the prone jackknife position. The internal opening was identified by injecting hydrogen peroxide (H_2_O_2_) into the external opening. If the internal opening could not be identified by injection of H_2_O_2_, its position was presumed based on the preoperative MRI. On the position over the internal opening, an intersphincteric curvilinear incision (1.5–2 cm) was made. After exposure of the intersphincteric fistula tract, it was severed adjacent to the internal anal sphincter. Then, the intersphincteric fistula tract was sharply dissected to the position traversing the external anal sphincter. The remnant tract was cored out from the external opening until to connect with the intersphincteric fistula tract. The defect on the external anal sphincter was repaired by suture with 3/0 Vicryl. A fan-shaped incision was made to excise the internal opening and an extremely small proportion of the internal anal sphincter. The remnant lateral full-thickness flap following the separation of the intersphincteric plane was sutured without tension to cover the repaired defect on the external anal sphincter. A radial wound on the distal anal canal was left for drainage (Figure 1).

#### Ligation of the Intersphincteric Fistula Tract

The LIFT was performed as previously described (21). Briefly, an intersphincteric curvilinear incision (1.5–2 cm) was made to expose the intersphincteric tract that was subsequently ligated adjacent to the internal and external anal sphincter, respectively. After a section of the intersphincteric tract was excised, the remnant tract was cored out from the external opening to the outer edge of the external anal sphincter. Finally, the intersphincteric wound was interruptedly sutured with 3/0 Vicryl.

#### Postoperative Management

Postoperatively, all patients received a soft diet and were prescribed an antibiotic and a stool softener for 1 week. Patients treated with SPF received sitz bath with traditional Chinese medicine, which was restricted in patients treated with LIFT. Dressings were performed with iodophor once a day in hospital and twice a week in the outpatient department after discharge.

#### Follow-Up

All patients were followed up in clinic or by phone at 3-month intervals. Clinical examination defined fistula healing, failure, or recurrence. Healing was defined as cicatrization of all wounds without discharge at 3 months. Failure was defined as persistence of any unhealed wound at 3 months or undergoing a fistulotomy due to connection between the intersphincteric wound and anal canal after LIFT. Recurrence was confirmed when an anal fistula or abscess was observed on any previously healed wound.

### Statistical Analysis

The SPSS for Windows version 22.0 (SPSS Inc., Chicago, Illinois, USA) was used to perform the statistical analysis. Numerical variables with a normal distribution were described as mean and SD, of which difference between groups was compared using the two-sample Student's *t*-test. Numerical variables with skewness distribution were expressed as median with range, of which difference between groups was compared using the Mann–Whitney *U*-test. The paired Student's *t*-test or Wilcoxon test was performed to compare the difference in numerical variables before and after operation. The Pearson's chi-squared test or continuity corrected chi-squared test was used to compare categorical variables, as appropriate. The Kaplan–Meier survival analysis was performed to assess the cumulative incidence of recurrence after the 2 procedures. A two-sided *p* < 0.05 was considered significant.

## Results

### Patient Characteristics

During the study period, 46 patients underwent SPF procedures, and 51 patients underwent LIFT procedures. In the SPF group, 5 patients with low transsphincteric fistulas were excluded. In the LIFT group, 13 patients with low transsphincteric fistulas and 3 patients with Crohn's disease were excluded. Eventually, 41 patients treated with SPF and 35 patients treated with LIFT were included for analysis.

The SPF group comprised 33 men and 8 women with a mean age of 36.6 ± 9.0 years, and 25 men and 10 women with a mean age of 35.0 ± 9.2 years were included in the LIFT group. Patients in the SPF group and in the LIFT group, respectively, underwent 43 SPF and 35 LIFT procedures. Three patients in the SPF group and 6 patients in the LIFT group underwent fistulotomy for additional intersphincteric fistulas on simultaneously. The median fistula length of 4 (range 3–10) cm in the SPF group was comparable with 4 (range 3–13) cm in the LIFT group. A majority of fistula internal openings in the LIFT group located in the anterior anal canal, but more internal openings in the SPF group located in posterior. The previous abscess incision and drainage and fistulectomy were performed on 1 and 3 patients in the SPF group, and on 2 and 2 patients in the LIFT group, respectively. Among them, 2 patients in the SPF group and 1 patient in LIFT group complained incontinence of flatus. There was no significant difference between two groups with regard to baseline characteristics including sex, age, fistula length and type, previous surgery, preoperative fecal continence function, and quality of life (Table 1).

**Table 1:** Baseline characteristics of patients.

**Item**	**SPF group (%)**	**LIFT group (%)**	***t*/*Z*/χ^2^**	***p***
Sex				
Male	33 (80.5)	25 (71.4)	0.857	0.354
Female	8 (19.5)	10 (28.6)		
Mean age (SD), years	36.6 ± 9.0	35.0 ± 9.2	0.759	0.450
Mean follow-up time (SD), months	11.4 ± 3.5	10.7 ± 4.3	0.774	0.441
Median length of fistula (range), cm	4 (3–10)	4 (3–13)	−0.357	0.721
Location of the internal opening				
Anterior	17 (39.5)	28 (80)	15.011	0.001
Lateral	9 (20.9)	5 (14.3)		
Posterior	17 (39.5)	2 (5.7)		
Intra-operative identification of the				
internal opening				
Yes	24 (55.8)	22 (62.9)	0.396	0.529
No	19 (44.2)	13 (37.1)		
Semi-horse fistula				
Yes	11 (25.6)	4 (11.4)	2.488	0.115
No	32 (74.4)	31 (88.6)		
Parks classification of fistula				
High transsphincteric tract	36 (83.7)	34 (97.1)	2.459	0.117
Suprasphincteric tract	7 (16.3)	1 (2.9)		
Previous history of operation				
Abscess incision and drainage	1 (2.4)	2 (5.7)	0.593	0.744
Fistulectomy	3 (7.3)	2 (5.7)		
No	37 (90.2)	31 (88.6)		
Fecal continence function				
Median Wexner score (range)	0 (0-2)	0 (0-1)	−0.463	0.643
Mean MRP (SD), kPa	14.7 ± 2.8	15.3 ± 2.8	−0.957	0.342
Mean MSP (SD), kPa	39.8 ± 4.0	40.7 ± 3.7	−0.997	0.322
FIQL score				
Lifestyle (range)	4 (3.8-4)	4 (3.9-4)	−0.463	0.643
Coping (range)	4 (3.8-4)	4 (3.8-4)	−0.448	0.654
Depression (range)	4 (4-4)	4 (4-4)	0.000	1.000
Embarrassment (range)	4 (3.7-4)	4 (3.7-4)	−0.113	0.910

*SD, standard deviation; MRP, maximum resting anal canal pressure; MSP, maximum squeeze pressure; FIQL, fecal incontinence quality of life*.

### Intraoperative Data

Intraoperatively, the intersphincteric tracts of 2 high-transsphincteric and 4 suprasphincteric fistulas, which were initially attempted at treatment with LIFT, were transected. This technical error that had been reported in our previous study resulted in the failure of LIFTs (21). As a remedy, the 6 patients switched to undergoing SPF.

### Comparison of Postoperative Outcomes Between SPF and LIFT Groups

Postoperatively, patients in both groups complained mild pain. There was no statistical difference in visual analog scale (VAS) scores on the 1st, 3rd, and 7th day between the two groups (Table 2). The mean follow-up periods were 11.4 ± 3.5 months in the SPF group, which was similar with 10.7 ± 4.3 months in the LIFT group. In the SPF group, 1 patient suffered secondary hemorrhage on the postoperative 5th day. Complications were recorded in 17 patients from the LIFT group, including dehiscence of the intersphincteric wound (*n* = 11) and abscess in the intersphincteric plane (*n* = 6). The dehiscent wounds all healed within 3 months after dressing. In 4 out of 6 patients with intersphincteric abscess, a connection between the intersphincteric plane and the anal canal was detected and treated by fistulotomy. The connection could not be found in the other 2 patients, and the wound healed after conservative treatment. Patients undergoing SPF had a significantly lower morbidity rate than patients undergoing LIFT.

**Table 2:** Clinical outcomes after surgery.

**Item**	**SPF group (%)**	**LIFT group (%)**	***t*/*Z*/χ^2^**	***p***
Postoperative pain				
Median VAS scores (range) on the 1st day	1 (1–3)	1 (1–2)	−0.220	0.826
Median VAS scores (range) on the 3rd day	2 (1–4)	2 (1–4)	−1.017	0.309
Median VAS scores (range) on the 7th day	0 (0–2)	0 (0–2)	−0.777	0.437
Overall complications	1 (2.3)	17 (48.6)	23.246	<0.001
Hemorrhage	1	0		
Dehiscence of the intersphincteric wound	0	11		
Abscess in the intersphincteric plane	0	6		
Mean wound healing time (SD), months	1.4 ± 0.3	1.7 ± 0.4	−3.381	0.001
Fistula healing				
Yes	42 (97.7)	27 (77.1)	6.084	0.014
No	1 (2.3)	8 (22.9)		

*SD, standard deviation; VAS, visual analog scale*.

**Figure 2:**
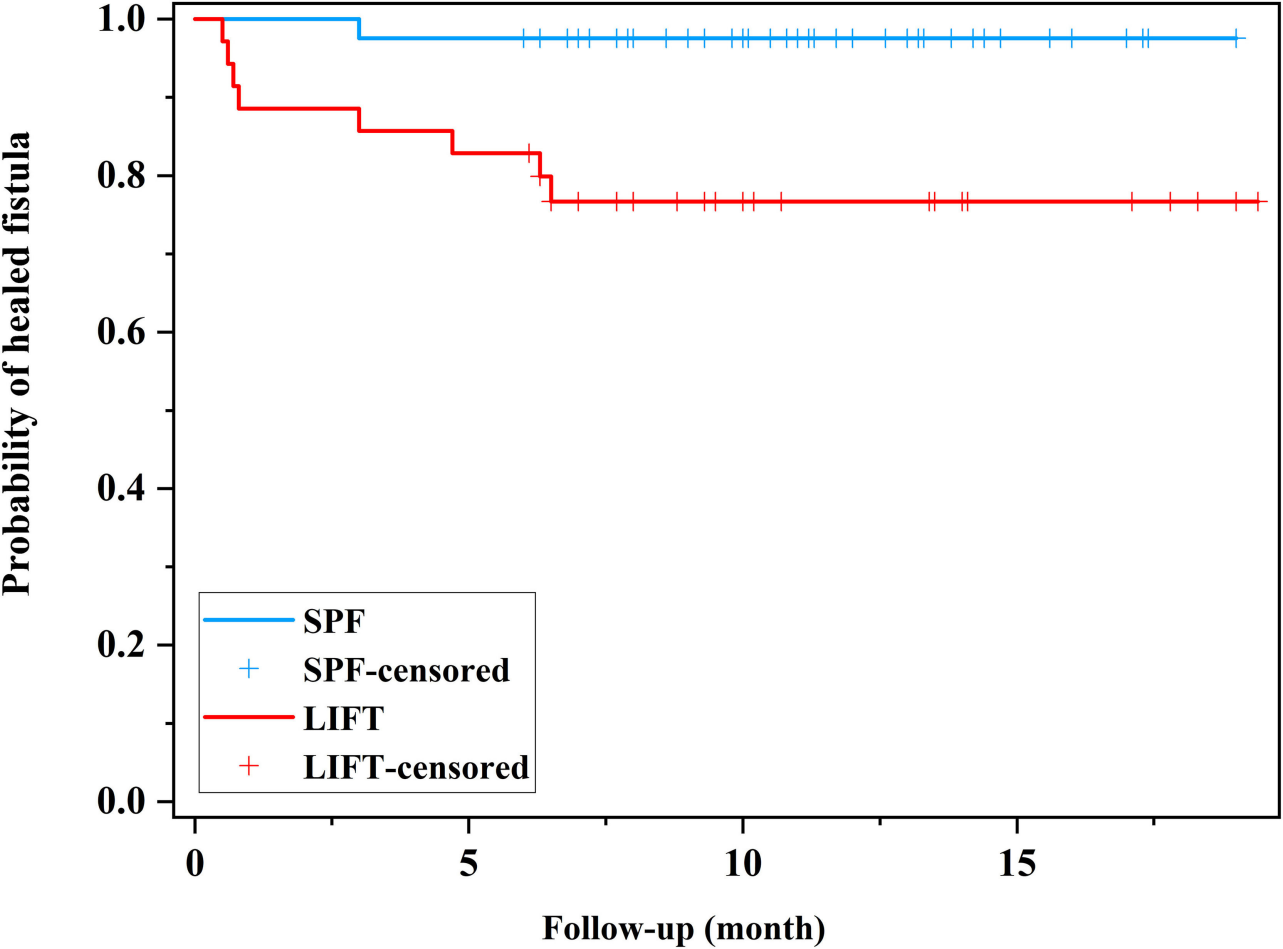
Kaplan–Meier curve showing the recurrence rate during the follow-up period. SPF, sphincter-preserving fistulectomy; LIFT, ligation of the intersphincteric fistula tract.

At 3 months, failure of wound healing after SPF happened in 1 patient with a suprasphincteric fistula, of which the intersphincteric tract cannot be clearly dissected. Likewise, the only one suprasphincteric fistula treated with LIFT remained unhealed at 3 months. Both the unhealed fistulas were treated with fistulotomy plus cutting-seton at 6 months. The mean time of wound healing was 1.4 ± 0.3 months in the SPF group, which was shorter than 1.7 ± 0.4 months in the LIFT group. At the end of follow-up, no patient suffered recurrence after SPF, whereas recurrence occurred in 3 patients treated with LIFT. The recurrent fistulas were all downstaged to intersphincteric fistulas. The survival analysis demonstrated a higher recurrence rate of LIFT than SPF over time (Figure 2). Eventually, the overall success rate of 97.7% of SPF was significantly higher than 77.1% of LIFT.

**Figure 3:**
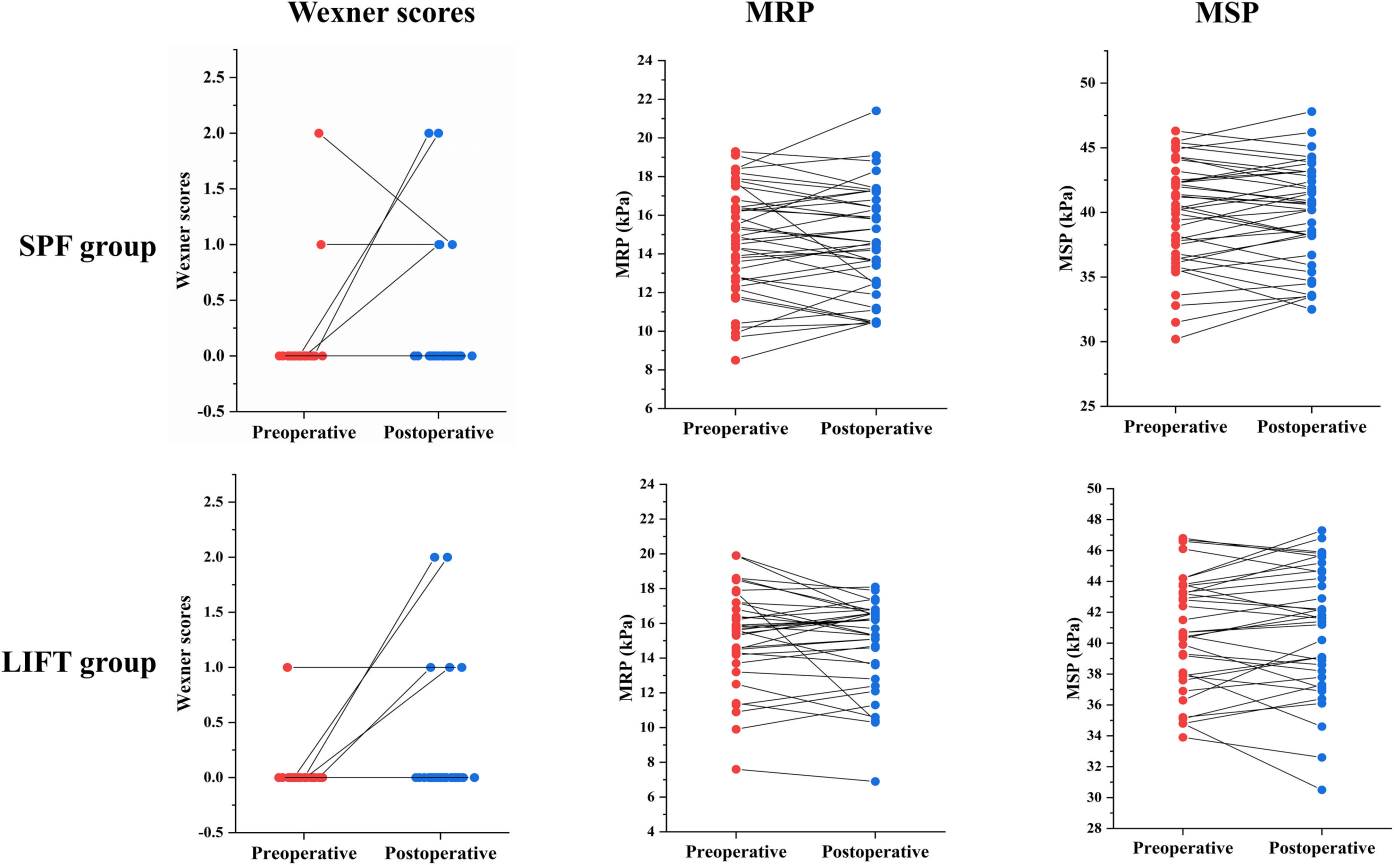
Change of Wexner scores, MRP, and MSP before and after surgery in the SPF and LIFT groups. MRP, maximum resting anal canal pressure; MSP, maximum squeeze pressure; SPF, sphincter-preserving fistulectomy; LIFT, ligation of the intersphincteric fistula tract.

In the SPF group, postoperative fecal continence function was improved in 1 patient with preoperative incontinence of flatus. Three patients undergoing SPF combined with fistulotomy complained new incontinence of flatus. Among them, 1 patient avoided eating outside, felt depressed, and ashamed. In the LIFT group, 4 patients undergoing LIFT combined with fistulotomy complained new incontinence of flatus. Among them, 1 patient felt unhealthy and ashamed. Another patient felt depressed. In both groups, there was no statistical difference in pre- and post-operative Wexner scores, MRP, MSP, and FIQL scores (Figure 3), which could also be found when comparing between two groups (Table 3).

**Table 3:** Outcomes of postoperative continence function and quality of life.

**Item**	**SPF group**	**LIFT group**	***t*/*Z***	***p***
Fecal continence function				
Median Wexner score (range)	0 (0–2)	0 (0–2)	−0.266	0.790
Mean MRP (SD), kPa	14.6 ± 2.8	14.9 ± 2.6	−0.469	0.641
Mean MSP (SD), kPa	39.9 ± 3.9	40.9 ± 4.1	−1.067	0.289
FIQL score				
Lifestyle (range)	4 (3.9-4)	4 (4-4)	−1.315	0.188
Coping (range)	4 (3.8-4)	4 (4-4)	−1.315	0.188
Depression (range)	4 (3.9-4)	4 (3.7-4)	−0.189	0.850
Embarrassment (range)	4 (3.7-4)	4 (3.7-4)	−0.113	0.910

*SD, standard deviation; MRP, maximum resting anal canal pressure; MSP, maximum squeeze pressure; FIQL, fecal incontinence quality of life*.

## Discussion

Management of a complex anal fistula remains a tricky problem, despite a variety of surgical procedures can be chosen. In the present paper, we reported a novel sphincter-preserving technique—SPF for the treatment of complex anal fistulas. The overall healing rate of fistulas after SPF was as high as 97.7%, with mild pain and negligible impact on fecal continence function.

The LIFT displayed a promising outcome for the treatment of complex anal fistulas, particularly transsphincteric fistulas. However, there is a room to improve the success rate of LIFT. Several additional procedures, such as AFP, platelet-rich plasma, and VAAFT, have been utilized to improve the fistula healing after LIFT (11–13). Their role is to eliminate the remnant tract instead of curettage, but the efficacy is limited. In clinical practice, we found that the treatment of the intersphincteric fistula tract had a greater impact on the outcomes. When treating high-transsphincteric or suprasphincteric fistulas with LIFT, it is difficult to integrally dissect and ligate the intersphincteric tract adjacent to the external anal sphincter due to the narrow and deep operating space, which may cause the failure of surgical operation. Therefore, in the current study, the majority of fistulas in the LIFT group located in anterior anal canal, because a shorter anal canal decreased the difficulty of LIFT operation. Correspondingly, the SPF procedure can integrally dissect the intersphincteric tract *via* cutting it off adjacent to the internal anal sphincter to increase operating space. Given that posterior transsphincteric fistulas treated by LIFT had a high recurrence rate of 71%, most posterior fistulas in this study were treated with SPF (22). It is believed that LIFT is technically demanding and difficult to perform in very high complex anal fistulas, especially for suprasphincteric and extrasphincteric fistulas (23). Correspondingly, 6 patients in this study suffered a technical failure of LIFT operation and then switched to undergoing SPF. This suggests that SPF can be performed as a rescue treatment after technical failure of LIFT.

Whether the internal opening is eliminated by LIFT remains controversial. It is generally believed that the internal opening is indirectly closed by LIFT adjacent to the internal anal sphincter. However, this kind of closure is unsecured. A connection between the intersphincteric space and the anal canal was found in some patients short after LIFT, which was treated with an unplanned fistulotomy and greatly decreased patient's satisfaction (21, 24). MAF was used to enhance the closure of the internal opening after LIFT. However, it did not increase the success rate of LIFT, instead increased the risk of complications (25). In addition, whether LIFT can eradicate the infected anal glands is another controversial problem. Removal of the infected anal glands is the essence in treating anal fistulas. LIFT can excise the infected glands locating in the intersphincteric space. In fact, most anal glands locate in the submucosa (26). It seems that LIFT has a defect in eradication of the infected glands, which may be one reason of a big difference in LIFT success rate from 37 to 87.65% (27, 28). In SPF procedure, a small fan-shaped incision was made to excise the internal opening and infected anal glands locating in submucosa or internal sphincter. In the SPF group, the only one unhealed suprasphincteric fistula had a very small intersphincteric fistula shown on MRI, and the internal opening could not be intraoperatively confirmed by injection of H_2_O_2_. These adverse factors led to a mistaken excision of the fibrotic muscle fiber as an intersphincteric tract. Unidentified internal opening and intersphincteric tract are considered as potential risk factors for SPF failure.

Wound dehiscence was the most common complication after LIFT (10). Insufficient drainage of collections may be the main cause. Although leaving the intersphincteric wound open may avoid this complication, it may also increase the risk of ligatures slipping off. As an improvement, the remnant lateral flap in SPF procedure was partially sutured, leaving a radial wound for drainage. In the SPF group, no patient suffered from wound dehiscence.

Several modified fistulectomy procedures have been demonstrated to have some potential in the treatment of complex anal fistulas. Fistulectomy plus primary sphincteroplasty procedure displayed a satisfactory success rate, but with postoperative wound dehiscence in 25% of patients, resulting in a keyhole deformity and fecal incontinence (17). It is essential to maintain the integrity of anal sphincter in treating complex anal fistulas. Core-out fistulectomy plus loose-seton procedure protected the integrity of anal sphincter (15). However, persistent open of the internal opening after seton removal may cause the fistula unhealing or recurrence. Compared with the two procedures, SPF can protect the integrity of anal sphincter, block the connection between the intersphincteric space and the ischioanal space *via* suture repair of the defect on the external sphincter, and enhance the strength of the repaired defect against high anal pressure by advancement of a lateral full-thickness flap. The anal morphology of patients was normal after SPF. New incontinence of flatus occurred in 3 patients with additional intersphincteric fistulas due to a more injury of the internal sphincter.

We recognize certain limitations to this study. As a retrospective study, inherent biases exist in reported data and assessment. Although preoperative MRI scans have been performed to identify the anatomy of fistulas, few postoperative MRI can be obtained to verify the radiological healing of fistulas. The sample size is too small for a univariate or multivariate analysis of risk factors. The follow-up period is short. A prospective study with a larger sample and a longer follow-up is needed to ascertain the long-term outcomes of SPF.

## Conclusions

Sphincter-preserving fistulectomy is a novel, safe, and effective sphincter-preserving technique for the treatment of complex anal fistulas, with a promising success rate and negligible impact on fecal continence. Additional prospective studies are warranted to evaluate the long-term outcomes of SPF.

## Data Availability Statement

The raw data supporting the conclusions of this article will be made available by the authors, without undue reservation.

## Ethics Statement

The studies involving human participants were reviewed and approved by Suzhou TCM Hospital Affiliated to Nanjing University of Chinese Medicine Institutional Review Board (No. 2017-006). Given the study's retrospective nature and data analyzed anonymously, it was exempt from obtaining informed consent from patients.

## Author Contributions

XW and XS designed the study. YH and ZX wrote the manuscript. YH, ZX, and YG collected, assembled, and analyzed the data. Project planning was performed by MS, YC, KW, XW, and XS. All authors read, edited, and approved the manuscript.

## Funding

This work was supported by grants from the Suzhou Society of Integrated Traditional Chinese and Western Medicine (SKJYD2021229) and the Suzhou Municipal Science and Technology Bureau (SKJY2021131).

## Conflict of Interest

The authors declare that the research was conducted in the absence of any commercial or financial relationships that could be construed as a potential conflict of interest.

## Publisher's Note

All claims expressed in this article are solely those of the authors and do not necessarily represent those of their affiliated organizations, or those of the publisher, the editors and the reviewers. Any product that may be evaluated in this article, or claim that may be made by its manufacturer, is not guaranteed or endorsed by the publisher.
